# Testing secondary sex ratio bias hypotheses in white‐tailed deer in Mississippi, USA

**DOI:** 10.1002/ece3.70296

**Published:** 2024-10-11

**Authors:** Jane E. Dentinger, Lacy A. Dolan Todt, Emma A. Schultz, James N. Helferich, Stephen Demarais, Randy W. DeYoung, William T. McKinley, Bronson K. Strickland, Melanie R. Boudreau

**Affiliations:** ^1^ Department of Wildlife, Fisheries & Aquaculture Mississippi State University Mississippi State Mississippi USA; ^2^ Caesar Kleberg Wildlife Research Institute, Texas A&M University‐Kingsville Kingsville Texas USA; ^3^ Mississippi Department of Wildlife Fisheries and Parks Jackson Mississippi USA

**Keywords:** Fisherian frequency dependence, local resource competition hypothesis, *Odocoileus virginianus*, Trivers–Willard hypothesis

## Abstract

Natural selection favors individuals with the highest inclusive fitness (i.e., total number of descendants). In cases where one sex is more productive, one or both parents may maximize their inclusive fitness by investing in the offspring of the more prolific sex. Such preferential production can lead to skewed sex ratios at various life history stages, including at birth, resulting in secondary sex ratio bias. Several competing hypotheses have been proposed to explain observed variation in secondary sex ratios including Fisher's frequency dependence and two hypotheses related to maternal condition: Trivers–Willard and the local resource hypotheses. Although it has been shown that maternal condition can influence the number of offspring produced in white‐tailed deer, there is no consensus as to which of the hypotheses drives sex ratio bias in wild populations. Using a spatiotemporally extensive dataset of pregnant white‐tailed deer from Mississippi, USA, we examined fetal sex ratio in relation to the Fisherian frequency‐dependence hypothesis and hypotheses related to maternal condition. While there was a male‐sex ratio bias in pregnant females that reduced in intensity with the number of offspring, there was no support for condition‐related hypotheses. Instead, secondary sex ratios for white‐tailed deer in Mississippi were nearly consistent with Fisherian frequency dependence. Our findings add to the body of literature on secondary sex biases in white‐tailed deer and help inform sex bias ratios for a southern population of a cervid of management importance in the US.

## Graphical abstract

We examine several competing hypotheses related to the variation in secondary sex ratios including Fisher's frequency dependence, Trivers–Willard, and the local resource hypotheses using a spatiotemporally extensive dataset of pregnant white‐tailed deer from Mississippi, USA. Our findings add to the body of literature on secondary sex biases in white‐tailed deer and help inform sex bias ratios for a southern population of a cervid of management importance in the US.

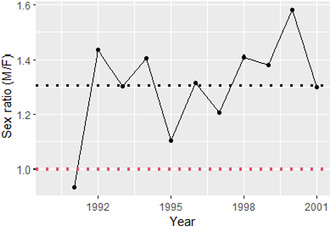

## 1 INTRODUCTION

Natural selection favors individuals that maximize their inclusive fitness by producing the most prolific and fecund offspring (Darwin, 1859). In species with two sexes, it is often assumed that offspring, regardless of sex, contribute equally to the inclusive fitness of their parents (Fisher, 1930). However, various types of mating systems such as polygyny, along with life history processes such as dispersal vary by sex, therefore, contributions to inclusive fitness by male and female offspring may not be equal. This disparity can lead to selective pressures that enact offspring sex ratio skews, of which there are many examples in wild populations (e.g., Douhard et al., 2019; Miquelle et al., 2015; Saragusty et al., 2009). This bias in the male‐to‐female offspring ratio may occur at conception (i.e., primary sex ratio, Hays et al., 2017), at the time of birth (i.e., secondary sex ratio, Rosenfeld & Roberts, 2004), in infancy (Torres & Drummond, 1997), or among breeding age adults (i.e., operational sex ratio, Carmona‐Isunza et al., 2017). Each type of sex ratio bias may have different causes and consequences for overall population structure. For example, bias in the primary sex ratio may indicate selective fertilization or implantation (Hewison & Gaillard, 1996), while bias in the operational sex ratio may indicate differential survival (Regan et al., 2020; Saunders & Cuthbert, 2015). These differences in sex ratio can cause changes to a population's growth trajectory, such as an increased proportion of females that could lead to greater population growth or an increase in males that may be the preferred harvested sex leading to population decline (Brook et al., 2000; Ginsberg & Milner‐Gulland, 1994; Underwood & Porter, 1985). Thus, a greater understanding of sex ratio bias can reveal important insights into underlying demographic processes and trends in free‐ranging animal populations essential for effective wildlife management.

There have been several competing hypotheses proposed to explain the drivers of secondary sex ratio bias. One of the earliest hypotheses, Fisherian frequency dependence, states that if offspring of both sexes contribute equally to parental fitness, as long as there is no operational sex ratio bias, then parental production of offspring should be equal between sexes (Fisher, 1930). However, if sex‐specific offspring production were to vary, the rarer sex would have a competitive advantage, incentivizing a return to a 1:1 equilibrium (i.e., frequency‐dependent selection; Fisher, 1930, Bull & Charnov, 1988). In populations with a consistent operational sex ratio bias, as is often the case for many large herbivore populations, there may be a correspondingly consistent, long‐term secondary sex ratio bias in the direction of the operational sex ratio bias. This produces a baseline shift from the 1:1 equilibrium (e.g., Milner et al., 2006). There is also some evidence that Fisherian frequency dependence may also act over shorter temporal scales such as within season (e.g., as in *Quiscalus quiscula* (Linnaeus), Common Grackles; Howe, 1977). For example, when a source of sex‐specific adult mortality (i.e., hunter harvest) aligns with the breeding season, there may be increased production of the rarer sex as the season progresses.

In the decades since Fisher first presented the frequency‐dependent hypothesis, many alternative hypotheses about secondary sex allocation have been proposed. Among those considered most plausible are two opposing hypotheses related to maternal condition. The Trivers–Willard hypothesis (Trivers & Willard, 1973) proposes that females in good condition should bias their offspring's sex ratio toward the sex with higher variation in reproductive success (i.e., a high‐risk, high‐reward strategy), whereas females in poor condition should favor offspring of the more reliably and consistently productive sex (Trivers & Willard, 1973; Veller et al., 2016). For polygynous mating systems, males are assumed to have higher variation in reproductive success (Bateman, 1948; Clutton‐Brock, 1988, 1989) with high‐quality males being able to appeal to many females resulting in the acquisition of more mating opportunities, while females produce offspring at a consistent but more limited rate. Thus, according to the Trivers–Willard hypothesis, females in good condition would be expected to produce more males, as male offspring in good condition would be more competitive, leading to bigger total reproductive payoffs (Røed et al., 2007). In contrast to the Trivers–Willard hypothesis, the local resource competition hypothesis states that female offspring are advantageous (Clark, 1978; Silk, 1983). Assuming that females are the more philopatric sex and that males tend to disperse from their natal ranges, adult females in areas of resource abundance should favor the production of daughters as their female offspring will inherit access to local resources, boosting their average reproductive success (Clark, 1978; Silk, 1983). Conversely, females in poor condition are expected to produce sons that disperse, mitigating local resource competition experienced by the mother (Clark, 1978; Silk, 1983).

Theories of sex allocation have been extensively studied in ungulates, as many species are both widespread and managed by recreational harvest, facilitating data collection. The white‐tailed deer (*Odocoileus virginianus* Zimmermann), a popular game species, is the most abundant and widespread herbivore in North America. While yearlings typically produce a single fawn, most adult females give birth to twins, although older deer in excellent body condition at conception may produce triplets, and occasionally quadruplets (Green et al., 2017) allowing for examination of within‐litter secondary sex ratios. Although, white‐tailed deer are polygynous (DeYoung et al., 2009), a key condition required for the Trivers–Willard hypothesis, studies of captive populations have found that females in better physical condition produce more female offspring, evidence that favors the local resource competition hypothesis (Ozoga & Verme, 1982; Verme, 1965, 1969). However, research on wild populations of white‐tailed deer have shown mixed results, with some studies supporting Trivers–Willard (Burke & Birch, 1995; Mansell, 1974; Woolf & Harder, 1979), other supporting the local resource competition hypothesis (DeGayner & Jordan, 1987; Verme, 1983, 1985), and some neither (Karns et al., 2014).

We used a spatiotemporally extensive data set to test hypotheses of secondary sex ratio allocation in white‐tailed deer in the southeastern United States. Our objectives were to investigate secondary sex ratio bias by first examining the average ratio of male‐to‐female offspring for females with single fawns, twins, or triplets. We then examined Fisherian frequency dependence at both coarse and fine temporal scales (i.e., across the year and within seasons), and the Trivers–Willard or the local resource hypotheses given indicators of the maternal condition. We predicted that if white‐tailed deer were operating under Fisherian frequency dependence there would be no change in the male‐to‐female sex ratio over time. We also predicted that if white‐tailed deer were operating under the Trivers–Willard hypothesis than greater maternal condition would result in more male offspring, while under the local resource hypothesis greater maternal condition would result in more female offspring.

## 2 MATERIALS AND METHODS

### 2.1 Study area

**FIGURE 1:**
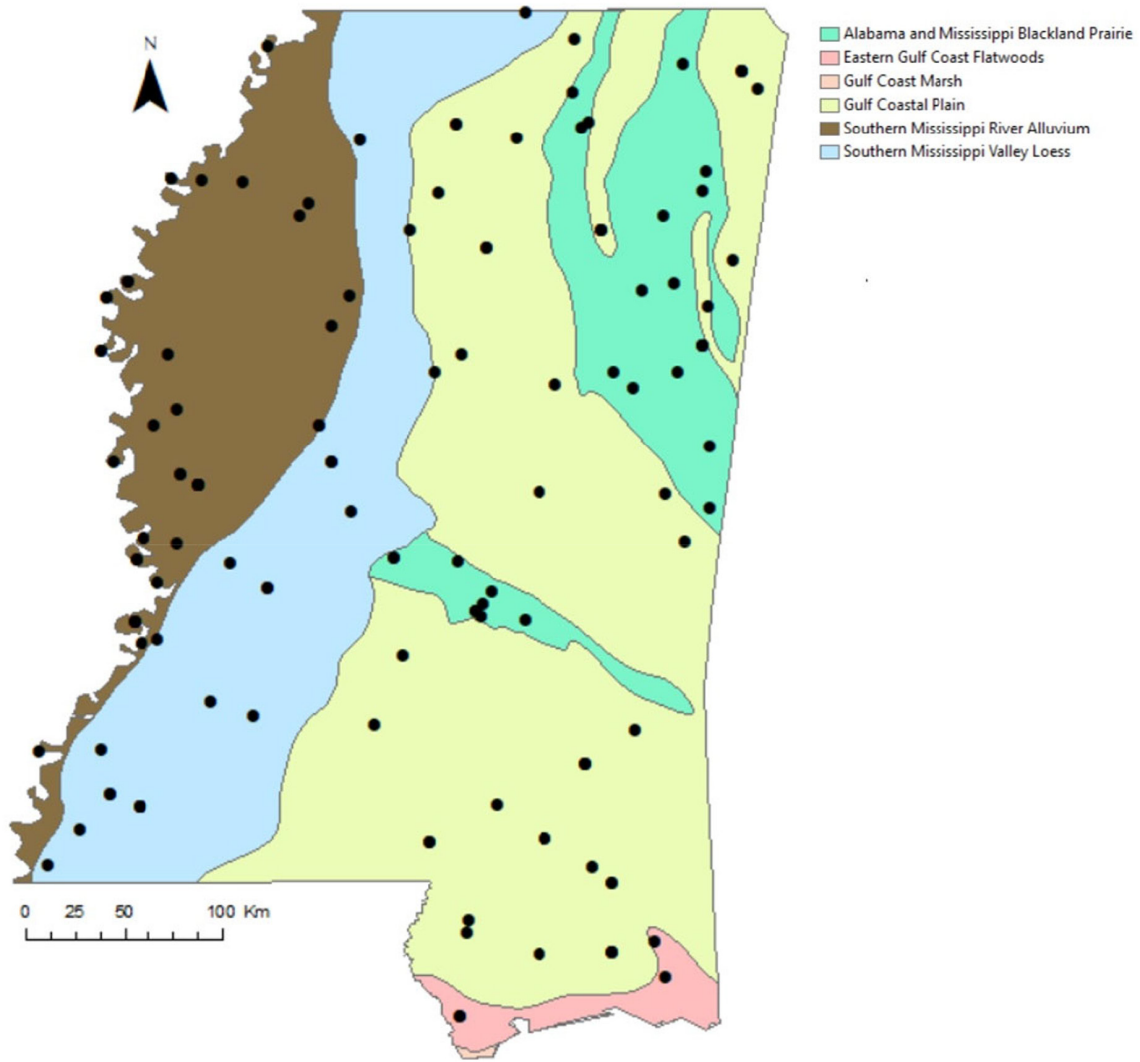
Sites where female white‐tailed deer, *Odocoileus virginianus* were harvested as part of annual herd health checks in Mississippi, USA from 1991 to 2001. Sites are overlain on soil regions for context.

White‐tailed deer were nearly extirpated from much of the southeastern United States by the early 1900s but recovered due to vigorous trapping and translocation programs (Warren, 2011). In Mississippi, white‐tailed deer were continuously distributed throughout the state by the 1990s, and as of 2023, the population is estimated to be at 1.75 million, with an annual statewide harvest of 280,000 individuals (Mississippi Department of Widlife, Fisheries [MDWFP], 2020, 2023). To ensure the continuation of this valuable resource, the MDWFP established an annual system of herd health checks to monitor disease as well as animal condition (e.g., Mississippi Department of Widlife, Fisheries, 2020). Health checks involved sharpshooting of 10 or more adult females per site on both private and public lands. From 1991 to 2001, data from health checks on white‐tailed deer were collected at 133 sites distributed throughout Mississippi (Figure 1). Mississippi is located in the humid subtropical climate region and is characterized by temperate winters, long and hot summers, and rainfall that is fairly evenly distributed throughout the year (Sherman‐Morris et al., 2012). Mississippi contains five major soil regions (Figure 1), each of which impacts the breadth of vegetation available to white‐tailed deer. Vegetation is dominated by oak (*Quercus* spp., Linnaeus) and several species of pines (*Pinus* spp., Linnaeus) in the southern part of the state, while fruit trees and hardwoods such as oaks and hickory (*Carya* spp., Nuttall) thrive in the north.

### 2.2 Deer data collection

Adult females were harvested from elevated stands adjacent to food sources or from vehicles at night with the aid of spotlights by MDWFP personnel (Demarais & Jacobson, 1982). While adult females may conceive in their first year (ca. 6 months of age) under excellent conditions most conceive at 1.5 years old (Green et al., 2017; Haugen, 1975). As a result, once a female familial social group (DeYoung & Miller, 2011) was spotted, personnel shot the first female in a group that appeared to be an adult (≥2.5 years old). Once harvested, age was estimated by tooth wear and replacement (Severinghaus, 1949). Adult female body condition was assessed using the eviscerated mass to the nearest 0.45 kg (i.e., dressed weight, hereafter “body mass”) and a kidney fat index (kidney fat divided by kidney mass; Finger et al., 1981). Collections were made as late as possible in gestation to facilitate identification of fetal sex (Strickland et al., 2011). When fetuses were removed, they were sexed and the conception date is estimated using forehead‐rump measurements (Hamilton et al., 1985). If a female had more than two fetuses that differed in size, the calculated conception dates were averaged.

### 2.3 Statistical analyses

**FIGURE 2:**
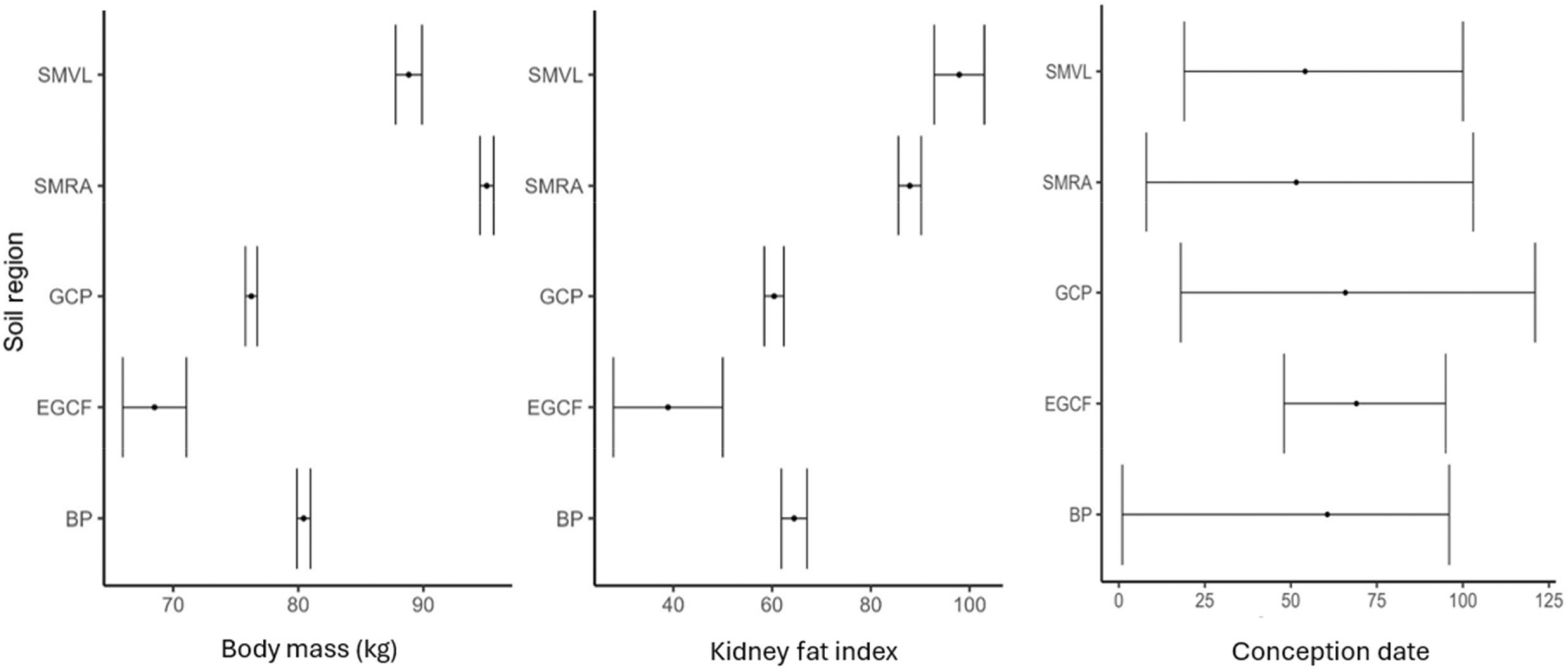
Average (±SE) body mass and kidney fat index and average (±range) conception date for white‐tailed deer, *Odocoileus virginianus*, in the five different soil regions of Mississippi, USA including the Southern Mississippi Valley Loess (SMVL), Southern Mississippi River Alluvium (SMRA), Gulf Coastal Plain (GCP), Eastern Gulf Coast Flatwoods (EGCF) and Blackland Prairie (BP), 1991–2001.

As older, larger‐bodied females with a greater amount of energy stores are likely to have more resources to invest in the production of young (e.g., Flajšman et al., 2017), we used body mass and kidney fat index as surrogate indices for body condition. To account for differences in local resource abundance that may impact condition across large geographic scales (McCullough, 1979, Figure 2), we assigned each adult female to one of five soil regions within Mississippi using the United States Department of Agriculture Major Land Resource Area shapefile (United States Department of Agriculture – Natural Resources Conservation Service, 2022; Figure 1) and adjusted body weight and kidney fat index by their regional means (value – regional mean) so that individuals in better condition had positive values, females in worse condition had negative values, and females at the regional average had a value of zero (McCullough, 1979). As there were no regional differences in conception dates, these were not adjusted (Figure 2).

We first examined the relationship between the ratio of male‐to‐female offspring (response variable) as a function of the number of offspring produced (predictor variable) using a linear model. Then, to assess how white‐tailed deer sex allocations changed in relation to Fisherian frequency dependance, we examined trends in the yearly average secondary sex ratio over our 12‐year study period. As yearly averages in secondary sex ratios were not temporally autocorrelated (Durbin–Watson test: *p* = .66), we used a linear model with sex ratio as the response variable and year as the predictor variable to determine if there was any measurable change in ratios over time or if they remained stable around an equilibrium point. We then further assessed if Fisherian frequency was occurring at a shorter within‐season time scale by examining the number of males produced (response variable) as a function of conception day (predictor variable). As the Julian day associated with conception spanned winter months, we adjusted values so that the earliest conception day (in November) was set to one, and all days thereafter were scaled to that starting point. As the relative value of females with one vs. two offspring is not directly comparable given the difference in maternal investment, we stratified the within‐season analysis data based on litter size (i.e., a single fawn, twins, or triplets). The single fawn model was fit using a binomial logistic regression (response variable, 0 = not male and 1 = male) with a logit link function. The twin and triplet models were fit using ordinal logistic regression (response variable, 0 = no males, 1 = one male, 2 = two males, 3 = three males) with the MASS R package (Venables & Ripley, 2002). To examine support for the Trivers–Willard or the local resource competition hypotheses, we examined the number of males produced (response variable) as a function of relative body mass and kidney fat index (predictor variables) using the same model structures as above (i.e., stratified by fetal number with logistic and ordinal logistic regressions). All analyses were performed in program R v. 4.2.2 (R Core Team, 2022).

## 3 RESULTS

Between 1991 and 2001, we obtained data from 2271 pregnant females. Of these, 1841 had complete records for body mass, kidney fat index, age, and region resulting in a total of 3260 fetuses, 39% of which were male (Table 1). As expected, most females were pregnant with twins, followed by females with a single fawn, and few females had triplets (Table 1). Data collected across years was uneven with fewer individuals collected in the earlier years and larger numbers in the later years (Table 1). Furthermore, collections were not equal among soil regions, with 20 deer collected in the Eastern Gulf Coast Flatwoods, 181 deer collected from the Southern Mississippi Valley Loess, 381 in Alabama and Mississippi Blackland Prairie, and 623 and 636 deer in the Southern Mississippi River Alluvium and Gulf Coastal Plain, respectively. The majority of collections occurred from February through March (94%) with occasional collections as late as June (0.3%). Across regions, conception dates were similar, with most conception dates occurring in December and January (Figure 2). Unadjusted body weights differed between soil regions with greater weights in Southern Mississippi River Alluvium and Southern Mississippi Valley Loess followed by Black Prairie, Great Coastal Plain, and the Eastern Gulf Coast Flatwoods (Figure 2) and a similar trend was seen in the kidney fat index (Figure 2).

**TABLE 1:** The number of offspring produced as single fawns, twins, and triplets, with the number of males in parentheses, across years for white‐tailed deer, *Odocoileus virginianus*, in Mississippi, USA, 1991–2001.

	1991	1992	1993	1994	1995	1996	1997	1998	1999	2000	2001	Total
Offspring from Single Fawns	7 (4)	9 (8)	15 (8)	45 (25)	29 (16)	70 (46)	53 (33)	56 (39)	49 (28)	67 (35)	73 (42)	473 (284)
Offspring from Twins	22 (10)	44 (24)	84 (48)	180 (108)	214 (113)	414 (230)	388 (207)	312 (177)	330 (190)	376 (239)	270 (153)	2634 (1499)
Offspring from Triplets	0	3 (1)	0	18 (5)	3	18 (9)	33 (17)	3 (1)	33 (21)	24 (12)	18 (9)	153 (75)
Total	29 (8)	56 (19)	99 (40)	243 (100)	246 (82)	502 (197)	474 (160)	371 (158)	412 (157)	467 (211)	361 (131)	3260 (1263)

**FIGURE 3:**
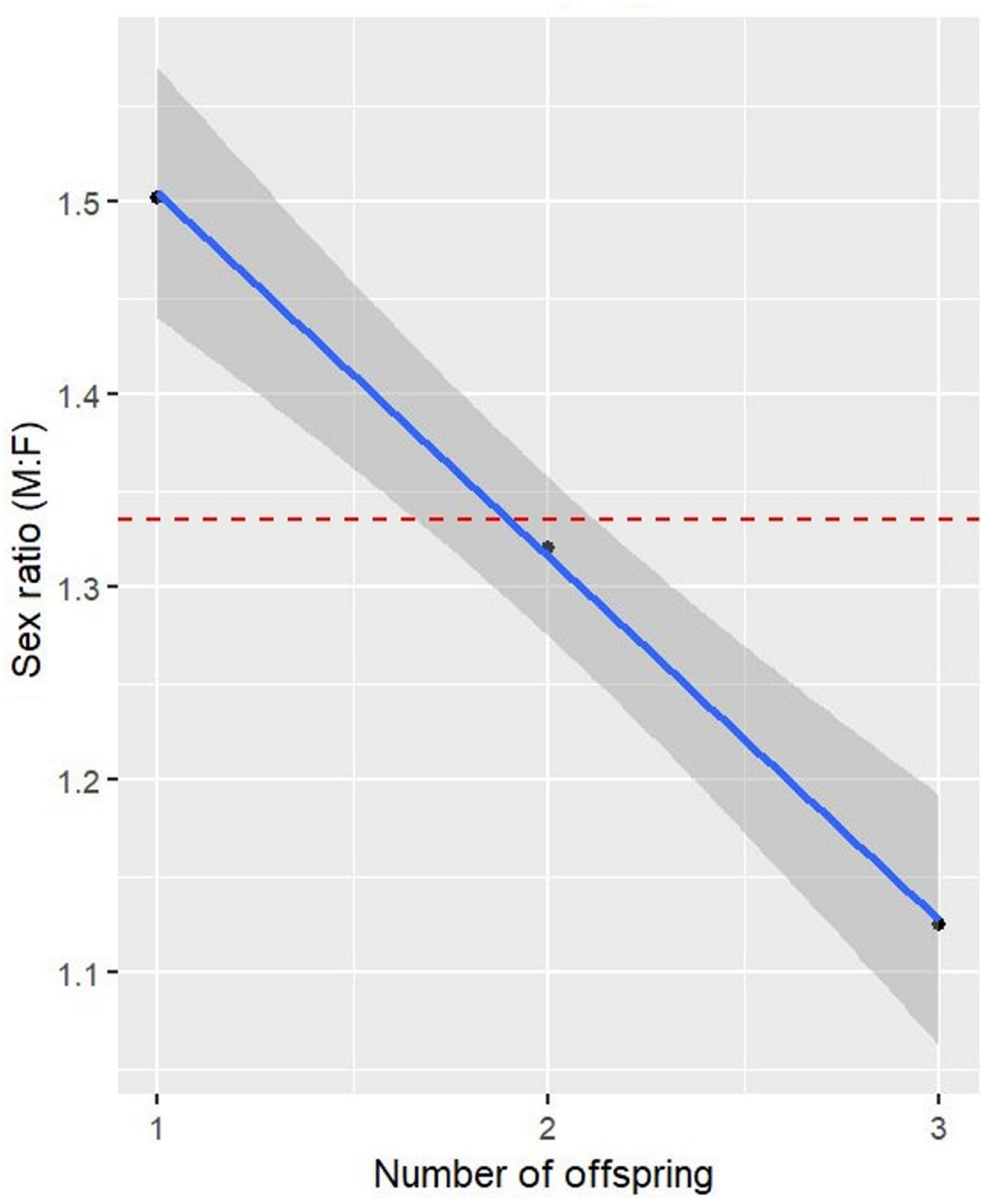
Male‐to‐female sex ratio in relation to the number of fetuses in a pregnancy. Data for white‐tailed deer, *Odocoileus virginianus*, in Mississippi, USA from 1991 to 2001.

**FIGURE 4:**
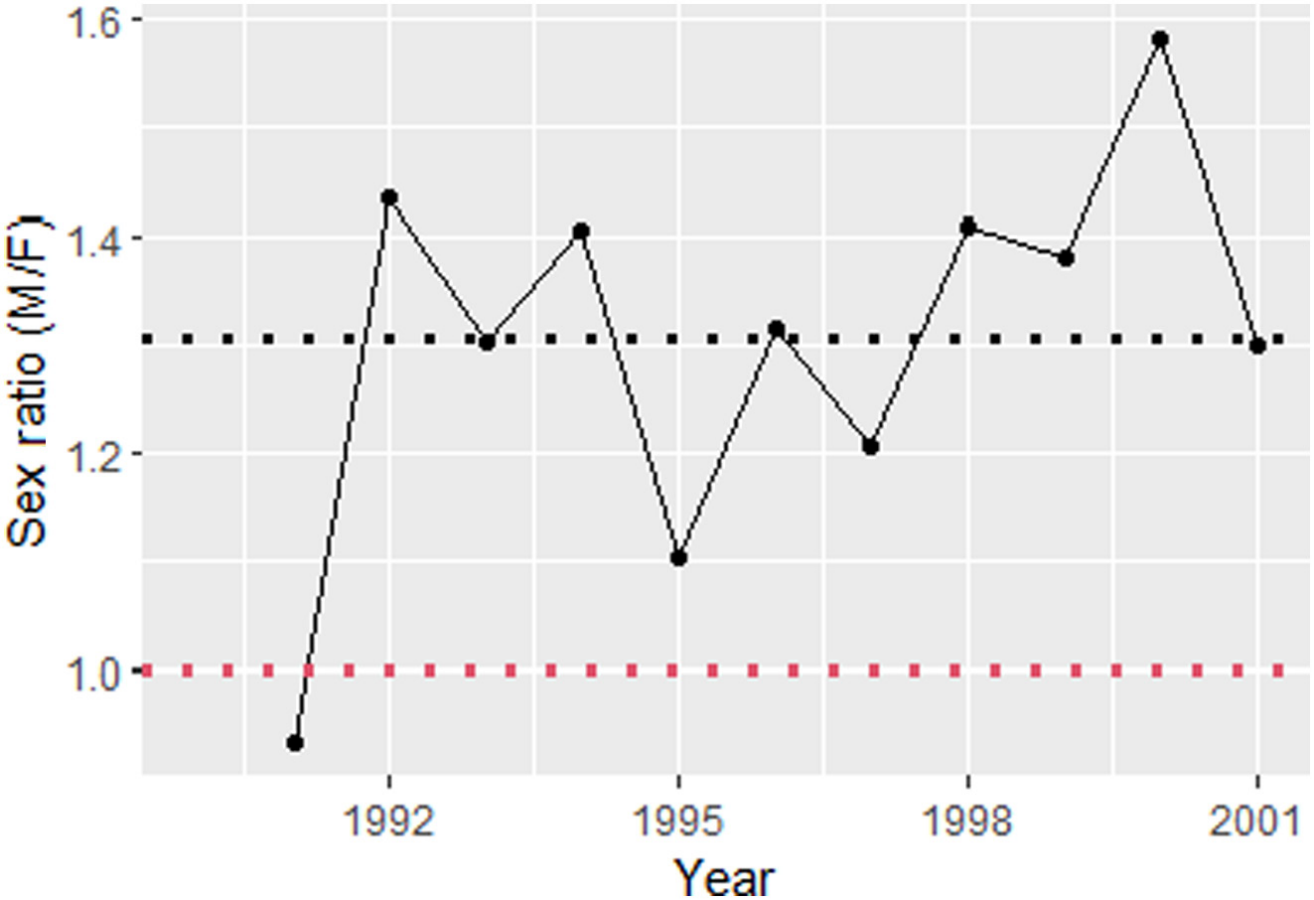
Yearly secondary sex ratios for white‐tailed deer, *Odocoileus virginianus*, in Mississippi, USA from 1991 to 2001. The average ratio (1.31) is indicated by a dotted black line and a ratio of (1:1) is indicated by a dotted red line.

**FIGURE 5:**
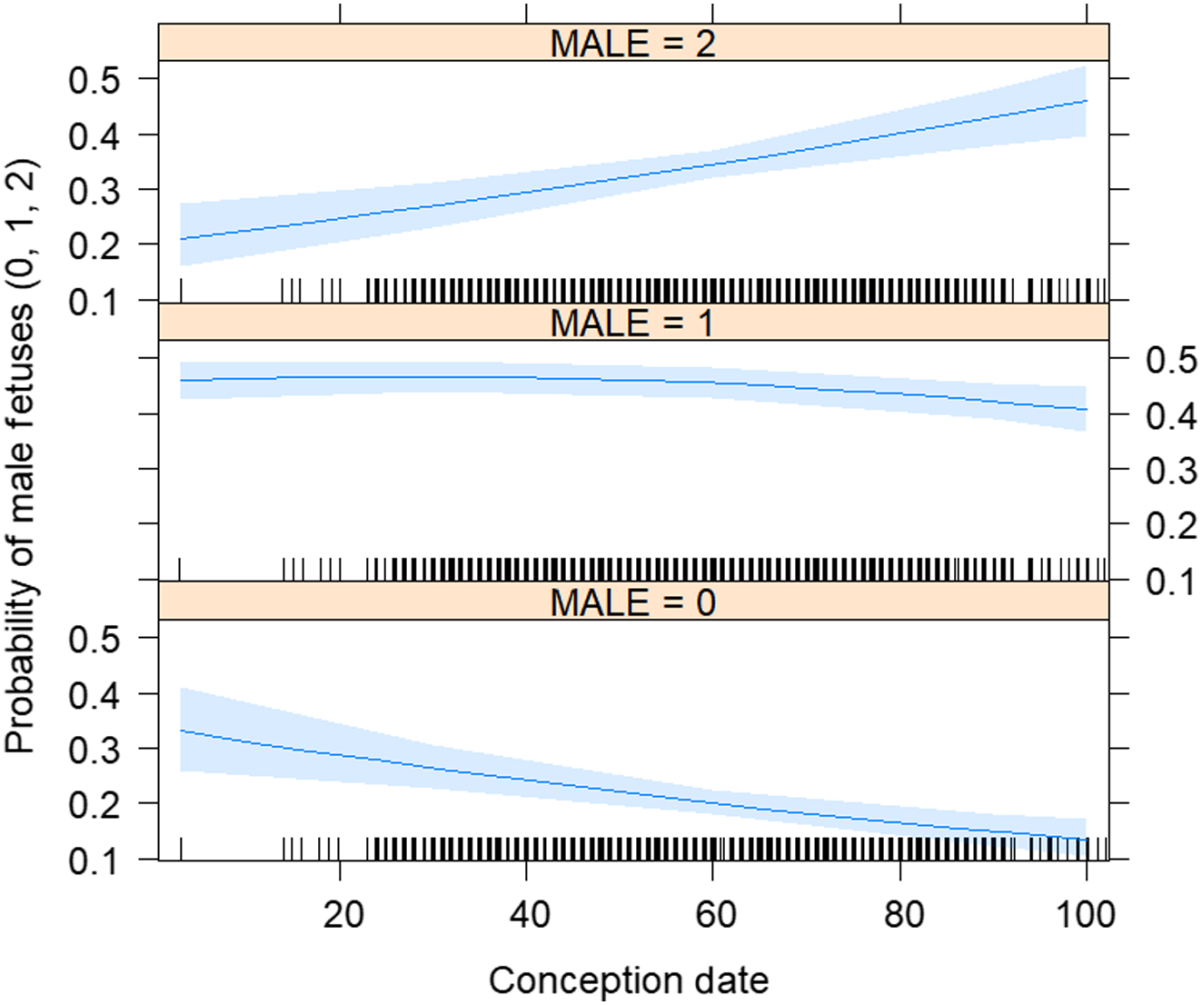
The probability of having a male offspring when pregnant with twins based on conception date for white‐tailed deer, *Odocoileus virginianus*, in Mississippi, USA from 1991 to 2001.

Females with a single fawn were most likely to be pregnant with a male (60%; 1.5:1 ratio), followed by females carrying twins (56%; 1.3:1 ratio), and then females with triplets (54%; 1.1:1 ratio) with sex ratio bias decreasing as the number of fetuses in a pregnancy increased (*β* ± SE = −0.19 ± 0.04; Figure 3). At the annual scale, we found that deer across Mississippi seemed to follow near Fisherian frequency dependence as sex ratios oscillated around a yearly baseline of 1.31 ± 0.05 (SE) males to females, with the slope of the relationship between sex ratio and time being nearly horizontal across years (β ± SE = 0.03 ± 0.02; Figure 4). At a within‐season time scale, there was no observable trend in the probability of having a male and date of conception for either single fawns or triplets. However, the number of males increased as the breeding season progressed given that a female was pregnant with twins, indicating that Fisherian frequency dependence may also be operating at shorter time scales under these conditions (Table 2, Figure 5). For the Trivers–Willard and local resource hypotheses, there was no indication that female condition (either relative body mass or kidney fat index) impacted the number of male offspring (Table 2).

**TABLE 2:** Beta‐coefficients with 95% confidence intervals for the relationship between the probability of producing male offspring and the conception date or indicators of body condition (i.e., body weight and kidney fat index) for females stratified by number of offspring per litter: A single fawn, twins, or triplets. Intervals that do not overlap zero are in bold. Data come from white‐tailed deer, *Odocoileus virginianus*, in Mississippi, USA, 1990–2001.

	Single fawn	Twins	Triplets
	β ± SE	β ± SE	β ± SE
Fisher's frequency dependance – seasonal scale			
Conception date	0.004 (−0.007, 0.015)	**0.01 (0.006, 0.018)**	0.02 (−0.011, 0.058)
Trivers – Willard vs. local competition			
Body mass	0.01 (−0.006, 0.027)	0.002 (−0.007, 0.011)	−0.003 (−0.043, 0.038)
Kidney fat index	−0.003 (−0.007, 0.001)	−0.001 (−0.003, 0.001)	0.01 (−0.001, 0.023)

## 4 DISCUSSION

We found that there was a relationship between the number of males as a function of the number of offspring produced, with the proportion of males becoming increasingly less skewed as the number of offspring increased a pregnancy. It is well established that there is a positive relationship between maternal age, body condition, and litter size in deer (Flajšman et al., 2017; Green et al., 2017; Johnstone‐Yellin et al., 2009) which is consistent with the large body of literature that supports the idea that older and larger‐bodied individuals produce more offspring due to an increased ability to allocate energy to reproduction when in better condition (Rollinson & Hutchings, 2013; Smith & Fretwell, 1974). However, among our metrics of body condition, we failed to find support for both the Trivers–Willard and the local resource competition hypothesis despite the fact that we had a robust sample size over both space and time. Failing to find support for either body condition hypothesis is not uncommon in mammals and has been observed in other white‐tailed deer populations (Diefenbach et al., 2019; Green et al., 2017; Karns et al., 2014). While this study adds to the lack of consensus on the driving mechanisms of secondary sex ratio bias among white‐tailed deer studies, there may be a variety of reasons for this effect. For example, compared with other cervids, white‐tailed deer occupy ecologically diverse landscapes across their range. In more northern climates (e.g., Canada), where the distribution and abundance of resources are less consistent, a reproductive strategy that maximizes the total rather than the type of offspring may be more beneficial to females, if the sexes have similar impacts on lifetime reproductive success. This is a strategy that has been seen in similar systems (e.g., Hagen et al., 2022; Stahlschmidt & Adamo, 2015). Additionally, due to the fact that deer populations tend to have a female‐biased operational sex ratio from generally lower male survival (even in unharvested populations), yearling males may have more breeding opportunities than would be traditionally expected (DeYoung et al., 2009). As a result, the potential offspring contribution among different sexes is perhaps less dramatic as would be required to produce results consistent with either the Trivers–Willard or the local resource competition strategies as compared with systems that have greater polygyny or where ungulates typically produce a single offspring. Thus, we posit that the wide geographic range of the species, along with potential variations in the intensity of polygyny, may contribute to the inconsistency in results across studies.

In contrast to our condition‐related hypotheses, at the annual scale, we found no change in sex ratio bias over time for white‐tailed deer in Mississippi, with values fluctuating around an average of 1.3 males to 1 female. While slightly higher than the perfect 1:1, this sex ratio is broadly consistent with male biased secondary sex ratios categorized in other harvested white‐tailed deer populations (1.09–1.13 males to 1 female in Pennsylvania; Diefenbach et al., 2019), and supports the idea that secondary sex ratios for white‐tailed deer in Mississippi act with near Fisherian frequency dependence. The probability of having a male fawn also increased later into the hunting season for pregnant females with twins implying that Fisher's frequency dependence may be also acting over smaller temporal scales, which is consistent with literature for other white‐tailed deer populations (Karns et al., 2014) and other taxa (Saalfeld et al., 2013). However, we did not see this temporal effect for single fawns or triplets, a result that may be constrained by the mechanism of the differential sex ratio production. Female deer typically ovulate between 1 and 3 eggs (Gastal et al., 2017), and while adult male deer produce male and female sperm equally (DeYoung et al., 2004), as far as we know females do not have any known mechanism by which to control sex ratio at conception. Thus, we assume sex‐biased selective abortion post‐conception is the only means by which to influence sex ratio, a mechanism which is not possible in females that ovulate a single egg or the near 40% of females that conceive multiple fetuses of the same sex. Additionally, triplet pregnancies were rare overall, potentially because few adult females are heavy enough to produce them and thus, the phenomenon may be driven by females with twins, and apparent only in this cohort at shorter timescales. While our results indicate Fisherian frequency dependence is likely occurring at various temporal scales for white‐tailed deer in Mississippi, further research concurrently measuring the secondary and operational sex ratios within populations would be needed for confirmation.

Information on white‐tailed deer sex ratios is important for land and wildlife managers as it can be used to assess demographic trajectories, inform adjustments to hunting policy, and be indicators of differences in deer ecology among regions of their range. Given that the breadth of existing studies on sex bias from cervid populations have shown mixed results (e.g., Borowik & Jędrzejewska, 2017; Caley & Nudds, 1987; Hewison & Gaillard, 1996; Kohlmann, 1999; Kucera, 1991; Macdonald & Johnson, 2008), we contend that sex ratio biases may be driven by a combination of factors, such as local densities, disturbances, and resources. However, density‐dependent effects on fecundity as a product of resource abundance may make disentangling the drivers of sex ratio bias from those acting on offspring numbers particularly difficult. If this is the case, then this has implications for management as increases in density may change production of both offspring number and sex ratios. If so, management could consider altering harvest rates as a function of density depending on management goals. Thus, our findings help inform sex bias ratios for the Mississippi population of a cervid of management importance in the US.

## AUTHOR CONTRIBUTIONS

**Jane E. Dentinger:** Conceptualization (equal); formal analysis (equal); investigation (equal); writing – original draft (equal); writing – review and editing (equal). **Lacy A. Dolan Todt:** Conceptualization (equal); investigation (equal); writing – original draft (equal); writing – review and editing (equal). **Emma A. Schultz:** Conceptualization (equal); investigation (equal); writing – original draft (equal); writing – review and editing (equal). **James N. Helferich:** Conceptualization (equal); investigation (equal); writing – original draft (equal); writing – review and editing (equal). **Stephen Demarais:** Conceptualization (equal); investigation (equal); methodology (equal); writing – review and editing (equal). **Randy W. DeYoung:** Conceptualization (equal); investigation (equal); methodology (equal); writing – review and editing (equal). **William McKinley:** Conceptualization (equal); funding acquisition (equal); investigation (equal); methodology (equal); project administration (equal); writing – review and editing (equal). **Bronson K. Strickland:** Conceptualization (equal); investigation (equal); methodology (equal); supervision (equal); writing – review and editing (equal). **Melanie R. Boudreau:** Conceptualization (equal); formal analysis (equal); supervision (equal); writing – original draft (equal); writing – review and editing (equal).

## CONFLICT OF INTEREST STATEMENT

The authors have no competing interests to declare.

## Data Availability

Data and associated data products are held by the Mississippi Department of Wildlife, Fisheries, and Parks and may only be available through a public records request. All code for analyses are located on Figshare: https://doi.org/10.6084/m9.figshare.c.7175712.
